# A practical and easy‐to‐scale protocol for removing chlorophylls from leaf extracts

**DOI:** 10.1002/aps3.70018

**Published:** 2025-07-29

**Authors:** Alice Fossati, Valeria Cavalloro, Daniela Rossi, Simona Collina, Emanuela Martino

**Affiliations:** ^1^ Department of Earth and Environmental Sciences University of Pavia Via Ferrata 1 Pavia 27100 Italy; ^2^ National Biodiversity Future Center Piazza Marina 61 Palermo 90133 Italy; ^3^ Department of Drug Science University of Pavia Via Taramelli 12 Pavia 27100 Italy

**Keywords:** chlorophylls, interferent removal, nature‐aided drug discovery, polyphenols, solid‐phase extraction

## Abstract

**Premise:**

Leaf extracts are valuable sources of bioactive compounds. However, co‐extracted chlorophylls interfere with analyses, including spectroscopic and biochemical assays. Existing methods for chlorophyll removal often have limitations, including the use of hazardous solvents, low specificity, or high costs.

**Methods and Results:**

A solid‐phase extraction protocol for chlorophyll removal from leaf extracts of *Corylus avellana* was developed using commercially available cartridges. The method requires standard equipment, can be completed within 10 minutes, and is scalable from analytical to preparative quantities. We validated this protocol across 20 taxa, demonstrating the removal of 85% of chlorophylls, successful scale up of quantities, cartridge reusability, and low solvent consumption.

**Conclusions:**

Key innovations of the protocol include simplified elution steps and the possibility of multiple reuse cycles. The simplicity, sustainability, and scalability of this new protocol make it particularly valuable for high‐throughput applications and process development.

Plants have always been used as sources of bioactive compounds, which are extracted to exploit their many positive effects on human health and well‐being (Newman and Cragg, 2020). Leaves are the most sustainable plant part from which to source bioactive compounds because their harvesting is not associated with the death of the whole organism (unlike roots or bark) and because they are easily available in large quantities. Many of the best‐known examples of plant bioactive compounds are extracted from leaves, including artemisinin, atropine, and camptothecin from *Artemisia annua* L., *Atropa belladonna* L., and *Camptotheca acuminata* Decne., respectively, to cite just a few (Markman, 1991; Weathers et al., 2011; Martino et al., 2017, 2019; Koetz et al., 2021).

However, during the extraction process, leaves release chlorophylls—green pigments found in abundance within leaves—as they are highly soluble in both nonpolar lipophilic and polar solvents (Kim et al., 2020). In a nature‐aided drug discovery (NADD) campaign, these pigments may alter the results of preliminary crude extract screening, leading to the incorrect selection of extracts for bioguided fractionation. In fact, due to their ultraviolet–visible (UV–Vis) absorbance between 400 and 700 nm and their ability to fluoresce in the red–far red range, chlorophylls can interfere with in vitro biochemical or enzymatic assays that rely on measurements of absorbance or fluorescence (Denizot and Lang, 1986; Carmichael et al., 1987; Donaldson, 2020; Lankatillake et al., 2021). Problems can also be encountered in cell‐based assays, in which these pigments can precipitate or undergo auto‐degradation due to light, oxygen, and water media, causing low reproducibility and accuracy (Oberhuber et al., 2003; Agarwal et al., 2014; Prinsloo et al., 2017; Samat et al., 2018). Moreover, the high‐intensity signals of chlorophylls may interfere with the analysis and quantification of analytes in mass spectrometry as a result of co‐elution with other metabolites (like carotenoids), their matrix effect (Van Nieuwerburgh et al., 2006; Bijttebier et al., 2014), or clogging of the high‐performance liquid chromatography (HPLC) apparatus.

The different types of chlorophylls comprise chlorophylls *a* and *b*, which are present in all oxygenic photosynthetic organisms, such as algae and plants; chlorophyll *c*, which is found in some species of algae; and finally chlorophylls *d*, *e*, *f*, and bacterio‐chlorophyll, which can be found in various algae (e.g., golden algae) and in some bacteria (Scheer, 2022). Chlorophylls *a* and *b* are of particular interest for a NADD approach as they are common in terrestrial plants and are composed of a hydrophobic phytol tail and a hydrophilic porphyrin head (Figure 1).

**Figure 1:**
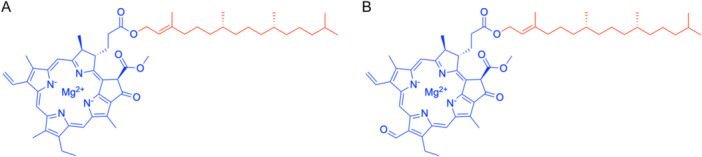
Comparison of chlorophyll *a* (A) and chlorophyll *b* (B) structures. The porphyrin head is shown in blue, and the phytol tail is shown in red.

Although extracts rich in these pigments have been developed for different purposes (Ebrahimi et al., 2023), chlorophylls are known to interfere with analytical and biological assays. Therefore, it is crucial to remove them from potentially active extracts before analysis and biological investigations. After their removal, chlorophylls can easily be recovered, dried, and re‐used as food supplements, colorants, and antioxidants in cosmetics and pharmaceuticals (Martins et al., 2023). The most widely used methods for chlorophyll removal are liquid–liquid extraction (LLE) or countercurrent separation (CCS). Both of these methods involve the use of nonpolar lipophilic solvents, which present several disadvantages (including being hazardous and not eco‐sustainable). For this reason, a number of alternative methods to remove chlorophylls from plants or algae extracts have been proposed. Some of these protocols involve the use of sulfuric and phosphoric acids, while others use sodium hydroxide to saponify chlorophylls, thus increasing their hydrophilicity to facilitate removal (Bahmaei et al., 2005; Li et al., 2016).

However, these methods may lead to undesirable reactions (e.g., hydrolysis, aldolic condensation, dehydration, retro‐aldolic cleavage, and isomerization reactions), as well as to the degradation or transformation of metabolites (Britton et al., 1995). A different approach for chlorophyll removal uses adsorbing materials, such as activated charcoal, activated bentonites, and strong ion‐exchange resins (i.e., AmberLite HPR900 OH; DuPont, Wilmington, Delaware, USA) (Chapman, 1994; Mokaya et al., 1994; Sabah, 2007; Makhoukhi et al., 2009; Shi et al., 2017; Liang et al., 2019; Phaisan et al., 2020; Jung et al., 2022; Vaz et al., 2022; Tang et al., 2023); charcoal, cellulose, and other polymers (Cardellina, 1983; Bijttebier et al., 2013; Tzima et al., 2020) are also available in packed cartridges for solid‐phase extraction (SPE). During the past decade, to aid in selective chlorophyll removal in dispersive solid‐phase extraction (dSPE), molecular imprinted polymers (MIPs)—synthetic polymers designed for a specific target molecule—have been developed (Batlokwa et al., 2013). While the selectivity of MIPs makes them a good alternative to SPE sorbents, their synthesis often requires environmentally harmful solvents, long reaction times (ca. 9 h), and costly starting polymers. Furthermore, they are not yet commercially available. Although dSPE can achieve results comparable to SPE when performed under similar conditions, it is not suitable for the parallel treatment of multiple samples due to its dispersive nature (Vergara‐Luis et al., 2023). The various drawbacks related to the different methodologies commonly used for chlorophyll removal are summarized in Table 1.

**Table 1:** The main techniques for removing chlorophylls and their relative drawbacks.

Technique	Drawbacks^a^	References
LLE/CCS	Use of hazardous and environmentally harmful solvents Multiple extractions Uses large amounts of solvents Batch conditions	Rawa‐Adkonis et al. (2006); Cuong (2020); Kim et al. (2020)
Saponification	Not selective Undesired isomerization of carotenoids or chemical reactions (aldol condensation, dehydration, retro‐aldol cleavage) Long saponification reaction times (30 min to 1 h) Use of hazardous and environmentally harmful solvents (petroleum ether and diethyl ether) Batch conditions	Granado et al. (2001); Larsen and Christensen (2005); Biehler et al. (2010)
Activated charcoal	Not selective Uses large amounts of solvents Difficult regeneration Batch conditions	Cooney et al. (1983); Raks et al. (2018); Tzima et al. (2020)
Activated bentonites	Difficult and long activation time (microwave activation or acid activation) Hazardous reagents Uses large amounts of solvents Difficult regeneration Batch conditions	Nebergall et al. (1994); Makhoukhi et al. (2009); Borah et al. (2022)
Resins	Low recovery Uses large amounts of hazardous solvents for resin regeneration Batch conditions High costs for resins ($60–$250 for 250 g)	Srivastava and Kuddus (2019); Vaz et al. (2022); Rosa et al. (2023)
MIPs	Expensive and difficult to produce Long synthesis time; requires hazardous solvents and not easily accessible prime materials Batch conditions	Adumitrăchioaie et al. (2018); Sajini and Mathew (2021); Kai et al. (2023)
dSPE	Not selective Batch conditions Solid phases can be expensive (from $200 to $500)	Tzima et al. (2020); Van der Vegt et al. (2022); Soares et al. (2023); United Chemical Technologies (2024)

*Note*: dSPE = dispersive solid‐phase extraction; LLE/CCS = liquid–liquid extraction/countercurrent separation; MIPs = molecular imprinted polymers.

^a^ Costs are given in U.S. dollars (USD) at the time of publication.

Cost and environmental considerations were taken into account throughout the development of this protocol in order to implement a cost‐effective and environmentally friendly procedure. *Corylus avellana* L. leaves were used to establish the protocol, and the optimized method was then applied to 20 leaf extracts obtained from different species to assess its robustness and versatility.

## METHODS AND RESULTS

### Development of the SPE chlorophyll removal protocol using *Corylus avellana* extract

Mature leaves of *Corylus avellana* were harvested within the Nature Reserve Bosco Siro Negri in Pavia, Italy (45°12′39″N, 09°03′26″E, 74 m a.s.l.). Leaves were dried in a drying room with active ventilation at 35°C until they reached a constant weight and then powdered using a blade mill (A10 IKA; Werke, Staufen, Germany). Extraction was performed by microwave‐assisted extraction in methanol (MeOH) and ethyl acetate (AcOEt), both of which are widely used for the extraction of different classes of metabolites from natural matrices, using a monomodal microwave oven (Discover LabMate Instrument; CEM Corporation, Matthews, North Carolina, USA) (Granata et al., 2021; Cavalloro et al., 2021, 2024). The extractions were carried out at 60°C, with 2 min of ramping and 5 min of heating, at 250 psi maximum pressure and with high stirring. For each extraction, 200 mg of pulverized leaves were suspended in 2 mL of solvent, with three total cycles of extraction and solvent renewal at each cycle. Chlorophylls *a* and *b* content of both extracts was measured using a UV spectrophotometer (FLUOstar Omega microplate reader; BMG Labtech, Ortenberg, Germany), following the method of Porra et al. (1989). Adsorption measurements were performed at 662 nm and 644 nm (*A*_662_ and *A*_644_, respectively), and chlorophyll concentration was calculated according to the following equations:

$$\begin{array}{ccl} \text{Chlorophyll }\text{a}\;(\mathrm{\mu g}/\text{mL}) & = & \left(9.78\times A_{662})-\;(0.990\times A_{644}\right) \end{array}$$

$$\begin{array}{lll} \text{Chlorophyll }\text{b}\;(\mathrm{\mu g}/\text{mL}) & = & \left(21.426\;\times \;A_{644})-\;(4.650\times A_{662}\right) \end{array}$$

This same method was used in all of the following steps of the protocol.

The results demonstrated that the methanolic extract contained 6.9 µg/mL of chlorophyll *a* and 6.1 µg/mL of chlorophyll *b*, while the AcOEt extract contained 14.7 µg/mL of chlorophyll *a* and 17.0 µg/mL of chlorophyll *b*. All the procedures described above (extraction with MeOH or AcOEt and chlorophyll quantification) were performed in triplicate. To develop the chlorophyll removal methodology, we started with the methanolic extract, as it is one of the most frequently used solvents in the early stage of the NADD process (Yao et al., 2004).

#### Solvent influence on purification

The methanolic extract was used to develop the chlorophyll removal procedure on a small scale (1‐cc cartridges). Among the commercially available SPE cartridges, we selected polymeric hydrophilic–lipophilic balance (HLB) cartridges, a reverse SPE constituted by two monomers: the hydrophilic *N*‐vinylpyrrolidone and the lipophilic divinylbenzene. This dual nature of the SPE absorbent allows interaction with both nonpolar and polar compounds, enabling this stationary phase to interact with both the hydrophobic phytol tail and the hydrophilic porphyrin head of chlorophylls (Figure 1). Moreover, because the cartridges are available in various sizes (bed mass from 30 to 500 mg) and as 96‐well plates, they can be used at different stages of drug discovery campaigns. To study the retention potential of the cartridges, dichloromethane (DCM), AcOEt, ethanol (EtOH), and MeOH were selected, keeping in mind their different polarity indices and their ability to solubilize chlorophylls. Waters OASIS HLB cartridges (1 cc; Waters Corporation, Milford, Massachusetts, USA) were mounted on the manifold and conditioned with 200 μL of the working solvent. Despite the cartridges being water‐wettable, it was not feasible to use 50% EtOH due to high back pressures and long elution times. The preliminary purification test was performed by loading approximately 6 mL of extract at 1 mg/mL, applying the vacuum, and washing the cartridge with another 6 mL of the same solvent. All the purified extracts were subjected to chlorophyll quantification, and the removal was evaluated by comparing the amount of chlorophyll in the untreated extract. DCM and AcOEt removed less than 30% of the considered pigments, whereas EtOH achieved purification of 65% and MeOH of almost 80% (Table 2).

**Table 2:** Percentage of chlorophyll removal depending on hydrophilic–lipophilic balance (HLB) cartridge size, loading capacity, and solvent.^a^

Cartridge size	Loading capacity	Solvent	Chlorophyll *a* removal (%)	Chlorophyll *b* removal (%)
1 cc	6 mL at 1 mg/mL	DCM	0%	8% ± 2.4
1 cc	6 mL at 1 mg/mL	AcOEt	22% ± 1.6	28% ± 1.3
1 cc	6 mL at 1 mg/mL	EtOH	65% ± 0.7	58% ± 1.1
1 cc	6 mL at 1 mg/mL	MeOH	76% ± 0.6	90% ± 0.5
1 cc	250 µL at 5 mg/mL	MeOH	92% ± 0.2	94% ± 0.3
1 cc	500 µL at 5 mg/mL	MeOH	85% ± 0.3	90% ± 0.1
1 cc	1 mL at 5 mg/mL	MeOH	67% ± 0.4	76% ± 0.5
1 cc	250 µL at 5 mg/mL	AcOEt	89% ± 0.6	93% ± 0.3
1 cc	500 µL at 5 mg/mL	AcOEt	66% ± 1.8	54% ± 0.9
6 cc	3 mL at 5 mg/mL	MeOH	93% ± 1.2	95% ± 1.1
6 cc	10 mL at 5 mg/mL	MeOH	92% ± 0.4	90% ± 0.2

*Note*: AcOEt = ethyl acetate; DCM = dichloromethane; EtOH = ethanol; MeOH = methanol.

^a^ All experiments were performed in triplicate.

#### Evaluation of the loading capacity of the cartridges

The results in Table 2 show that MeOH is effective for chlorophyll removal. In the first set of experiments, the cartridge loading volumes were evaluated, considering extracts obtained using MeOH as extractive solvent. Different loading volumes (250 µL, 500 µL, and 1 mL at 5 mg/mL in MeOH) were tested, followed by vacuum application and cartridge washing with the same volume of MeOH. Pigments were quantified after the purification process (Table 2). While cartridges loaded with 250 μL or 500 μL of extract were similarly efficient (Welch's *t*‐test, *P* = 0.2120, non‐significantly different, experiments performed in triplicate), a loading capacity of 500 µL was determined to be the best option because it allows for the simultaneous processing of a larger quantity of extracts. The purification process was monitored using thin‐layer chromatography (TLC). The first TLC method applied (method A: 6 hexane/1 AcOEt/1 acetone/0.4 MeOH; detection with a UV lamp [Desaga MinUVIS; Sarstedt‐Gruppe, Nümbrecht, Germany] at 366 nm) demonstrated a noticeable reduction in chlorophyll content in treated extracts compared to untreated extracts when observed at 366 nm, confirming their removal (Agarwal et al., 2014). Application of an orthogonal TLC method (method B: 4 toluene/3 AcOEt/3 MeOH/0.1 formic acid; detection under UV light at 254–366 nm, and 0.2% 2,2‐diphenyl‐1‐picrylhydrazyl [DPPH] in MeOH stain) showed that the anti‐scavenging activity of the extracts was not affected by the chlorophyll removal process (Appendix S1; see Supporting Information with this article). The developed method was then applied to the dried extract obtained using AcOEt as an extractive solvent, which was characterized by a high chlorophyll content (Table 2). As expected, the efficiency of pigment removal was lower compared to the methanolic extract. When using different solvents, it is advisable to quantify chlorophylls beforehand to adjust cartridge loading, ensuring an optimized removal process.

#### Extract analysis

**Figure 2:**
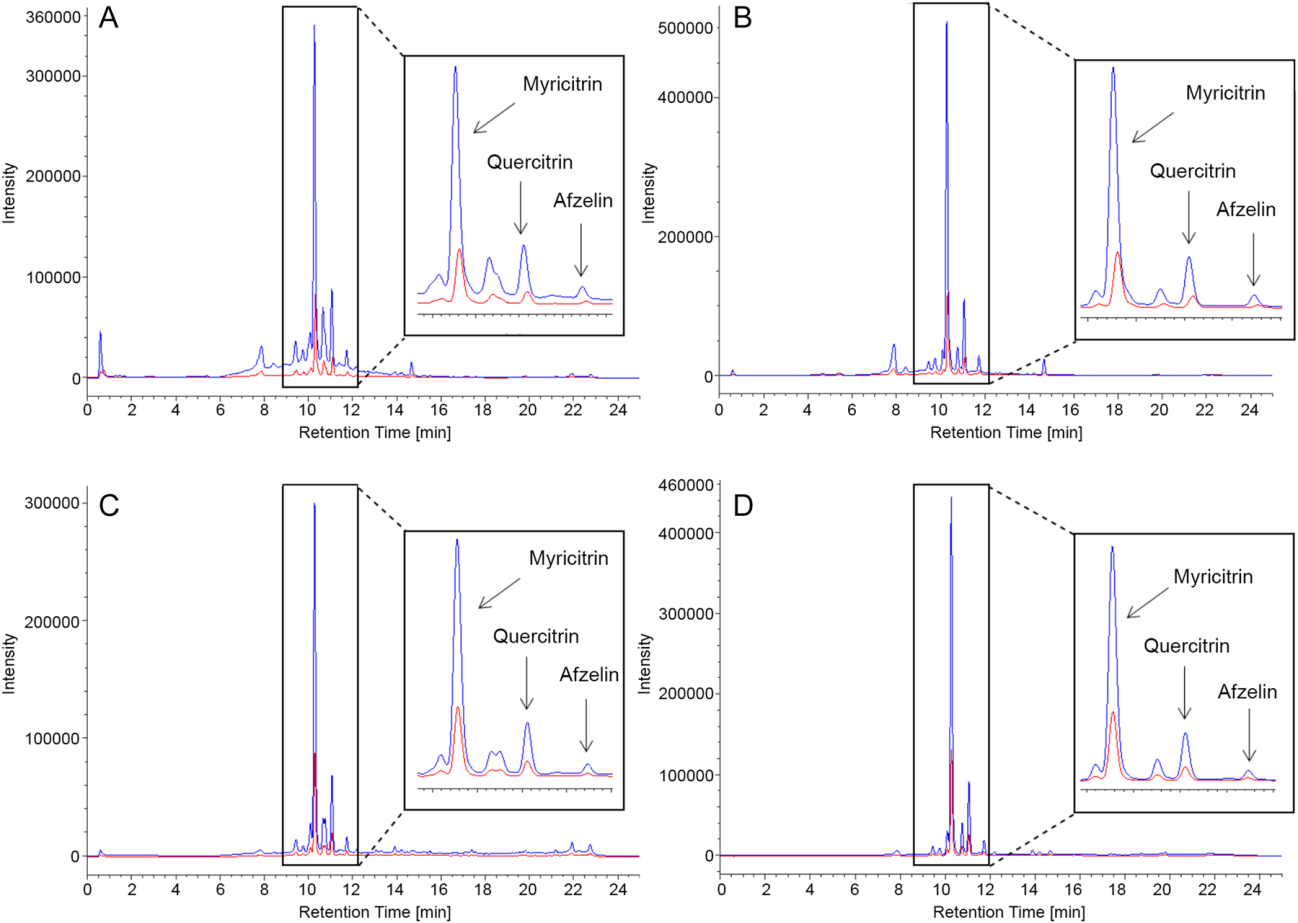
Chromatograms (λ = 320 nm) of *Corylus avellana* extracts before (red) and after (blue) the solid‐phase extraction (SPE) treatment under optimized conditions. (A) Ethyl acetate (280 nm), (B) ethyl acetate (320 nm), (C) methanol (280 nm), (D) methanol (320 nm). High‐performance liquid chromatography (HPLC) analyses were carried out using a Waters Acquity BEH C18 (100 × 2.1 mm, 1.7‐µm pores), with a flow rate of 0.350 mL/min and injection volume of 10 µL (Commisso et al., 2019). Prior to the injection, the extracts were filtered with 0.45‐µm nylon filters (PerkinElmer Inc.).

To evaluate the selectivity of our purification protocol, the methanolic extracts (both before and after SPE) were analyzed using an HPLC‐UV/photodiode array detector (DAD). First, the suitability of a previously developed HPLC method (Commisso et al., 2019) was evaluated by analyzing the *C. avellana* extract and identifying its main peaks as compared to quercitrin, myricitrin, and afzelin used as analytical standards (Figure 2).

MeOH was removed by evaporation under vacuum from both extracts (both before and after SPE treatment) until they were dried. The dried extracts were then re‐solubilized in MeOH at the same concentration and analyzed by TLC, HPLC‐UV/DAD, and HPLC–mass spectrometry (HPLC‐MS). The results demonstrated that chlorophyll removal preserved the qualitative analytical fingerprint of the crude extracts. Comparison of the LC‐UV/DAD signal intensity of the main metabolites revealed that the treated extract was approximately three times richer in the considered metabolites compared to the untreated one.

#### Scaling up the method and cartridge recycling

With the aim of making the newly developed method flexible and applicable to larger amounts of extract, the procedure was then scaled up from 1‐cc to 6‐cc HLB cartridges, loading an increased volume of extract, from 3 mL to 10 mL (5 mg/mL, in MeOH). The results obtained from three independent experiments (data not shown) demonstrated that the efficiency in chlorophyll removal is maintained up to 10 mL of extract.

Next, to develop an eco‐sustainable chlorophyll removal method and to try to minimize its environmental impact, the possibility of recycling the cartridges was evaluated. First, the use of acetone (a nonpolar solvent listed as environmentally safe) to regenerate the cartridges was tested. Next, five additional chlorophyll removal processes were tested on *C. avellana* methanolic extract, using both 1‐cc and 6‐cc cartridges, washing the cartridges with acetone and quantifying chlorophylls between each cycle. The results demonstrated that, despite a 10% loss in efficiency, the overall efficacy of chlorophyll removal remained satisfactory up to the fifth SPE cycle, reaching chlorophyll removal higher than 80%, with cartridge washings between each cycle (Table 3). The chlorophyll removal percentage was calculated by applying Porra equations to quantify chlorophylls at each cycle and comparing their amounts to the chlorophyll content of the starting crude extract. The washing fractions were analyzed via both TLC and HPLC, and no peaks different from chlorophylls were detected.

**Table 3:** Percentage of chlorophyll removal after five solid‐phase extraction (SPE) and washing steps.^a^

SPE purification cycle	Chlorophyll *a* removal (%)	Chlorophyll *b* removal (%)
1	92%	90%
2	85%	85%
3	83%	82%
4	80%	80%
5	80%	80%

^a^ Performed as a single experiment.

#### Comparison with conventional methods

The results obtained using the above methods were compared to those obtained using two diffused conventional methods, dSPE with charcoal (Amri et al., 2017) and LLE with *n*‐hexane (Tzima et al., 2020). For the dSPE method, two cycles using renewed charcoal were necessary, as the first cycle removed less than 70% of chlorophylls. The results obtained with the dSPE method were similar to those obtained with the newly developed method, while LLE with *n*‐hexane was less efficient (Table 4). However, dSPE required the use of 20% (w/w of extract) of charcoal, and as, to the best of our knowledge, no useful protocol for charcoal regeneration exists, it could not be recycled. The LLE method, in addition to being less efficient, uses hazardous solvents such as *n*‐hexane. Furthermore, both of these techniques required from 30 min (dSPE) to 1 h (LLE), whereas the newly developed method took only 10 min including the solubilization of the sample, equilibration of the cartridge, and SPE. The full protocol is described in Appendix 1. Moreover, the newly developed method allows parallel treatment of multiple samples. In fact, with SPE, multiple cartridges can be mounted on the same manifold and loaded with different extracts, allowing for their simultaneous purification. Although multiple purifications can be set up with dSPE and LLE using different extracts, only one extract can be processed at a time.

**Table 4:** Percentage of chlorophyll removal of the scaled‐up procedure compared with two diffused conventional methods.^a^

Method	Chlorophyll *a* removal (%)	Chlorophyll *b* removal (%)
6‐cc HLB cartridge	92% ± 0.4	90% ± 0.2
dSPE with charcoal (1 cycle)	68% ± 1.6	70% ± 1.1
dSPE with charcoal (2 cycles)	95% ± 2.7	94% ± 2.3
LLE with *n*‐hexane	72% ± 0.5	84% ± 0.7

*Note*: dSPE = dispersive solid‐phase extraction; HLB = hydrophilic–lipophilic balance; LLE = liquid–liquid extraction.

^a^ All experiments were performed in triplicate.

### Application of the developed procedure to extracts from 20 plant species

Because the aim of this study is removing chlorophylls without altering the profile of secondary metabolites, primary metabolites such as amino acids, lipids, and vitamins were not considered. To monitor the profile of secondary metabolites, TLC, HPLC‐UV/DAD, and HPLC‐MS were selected as analytical techniques, as they are the most common methods applied for extract analysis. Nevertheless, because secondary metabolites with different polarities can behave differently, slight ad hoc modifications of the protocol may be necessary across varying metabolite profiles.

To address this analytical challenge and evaluate the wide applicability of the protocol, we applied the newly developed method to 20 species that are part of a wider ongoing project aimed at biodiversity valorization—the National Biodiversity Future Center (NBFC) (https://www.nbfc.it/). Based on phylogenetic considerations, almost 700 species were selected, from approximately 12,000 species growing in Italy, as representative of the considered flora for sampling. Methanolic extract (provided by the University of Verona) of each of the following 20 selected plant species was investigated: *Adenophora liliifolia* (L.) A.DC., *Allium angulosum* L., *Allium lusitanicum* Lam., *Aloysia citrodora* Paláu, *Althaea officinalis* L., *Aquilegia atrata* W.D.J.Koch, *Beta vulgaris* L., *Carissa macrocarpa* (Eckl.) A.DC., *Castanea sativa* Mill., *Dianthus superbus* L., *Echium vulgare* L., *Eryngium maritimum* L., *Gratiola officinalis* L., *Inula salicina* L., *Lythrum salicaria* L., *Petasites paradoxus* (Retz.) Baumg., *Russelia equisetiformis* Schltdl. & Cham., *Salvia pratensis* L., *Succisa pratensis* Moench, and *Typha laxmannii* Lepech. Examples of the resulting metabolite profiles obtained from this analysis include: *Althaea officinalis* contains several classes of secondary metabolites, including anthraquinones, cardiac glycosides, lignins, phlobatannins, tannins, terpenoids, sterols, and steroids (Farhat et al., 2022); *Aloysia citrodora* contains iridoid glycosides, lignans, monoterpens, monoterpenoids, sesquiterpens, sesquiterpenoids, norisioprenoids, and naphtoquinones (Bahramsoltani et al., 2018); and *Gratiola officinalis* contains jasmonates, cucurbitane‐triterpene glycoside, and lupane‐type triterpenic acids (Bianchini et al., 2025). The diversity of these metabolites across the selected species may further support the broad applicability of our method.

All the methanolic extracts were subjected to our chlorophyll removal procedure. Their chromatographic fingerprints were developed using TLC and HPLC‐DAD (Appendices S2–S5). The efficiency of the chlorophyll removal process consistently exceeded 85%, for both chlorophyll *a* and chlorophyll *b* (Appendix S6). The chemical integrity of the extracts following SPE treatment was validated through complementary chromatographic techniques, using both TLC and HPLC‐UV/DAD. The comparison of the chemical fingerprints of all extracts across multiple detection wavelengths before and after SPE treatment confirmed that the procedure preserved the composition of secondary metabolites in the extracts. These results across 20 diverse plant species provide a strong indication of the method's broad applicability. However, this pilot study cannot guarantee universal effectiveness, and we recommend a case‐by‐case analysis of extracts before and after SPE treatment when applying this method to new species to avoid any undesirable loss of relevant compounds.

The investigation was extended to the evaluation of total phenolic content (TPC) and antioxidant activity, again using *C. avellana* methanolic extract for initial testing (Appendix S6). The TPC of the before and after SPE extracts was assessed spectrophotometrically following a previously developed micronized method (Attard, 2013). A gallic acid calibration curve was constructed, and six solutions of gallic acid in MeOH, at different concentrations (0.009–0.094% [w/v]), were prepared and seeded in triplicate. The same procedure was applied, also in triplicate, for the analysis of *C. avellana* methanolic and AcOEt extracts at the concentration of 5 mg/mL in MeOH. The phenolic values reported here are expressed as gallic acid equivalents (μg/mL). As postulated, SPE treatment led to an enhancement of TPC; this was more evident in the AcOEt extract, which was originally richer in chlorophylls (methanolic extract TPC 16.7–18.2 μg/mL gallic acid equivalents [GAE]; AcOEt extract TPC 18.0–33.6 μg/mL GAE). Therefore, chlorophyll removal also led to extracts with a higher concentration of metabolites, specifically phenols, characterized by antioxidant properties.

Based on the above considerations, the antioxidant activity of purified extracts was evaluated using a previously developed UV–VIS DPPH assay (Pellavio et al., 2017), which was micronized to be used with a microplate reader to save reagents, samples, and analysis time (Pegan et al., 2010; Blass, 2015; Jaganjac et al., 2021). The results showed that the antioxidant activity of the *C. avellana* extracts was higher after the chlorophyll removal step, increasing from 92% to 94% for the methanolic extract and from 79% to 83% for the AcOEt extract (experiments were performed in triplicate). It can be seen that the increase in antioxidant activity was more significant in the AcOEt extract, being originally richer in chlorophylls. This finding suggests that chlorophylls may be considered diluents of active ingredients, as evidenced by the increased percentage of active metabolites in extracts after SPE treatment versus before SPE treatment, even when the concentration remains the same.

**Figure 3:**
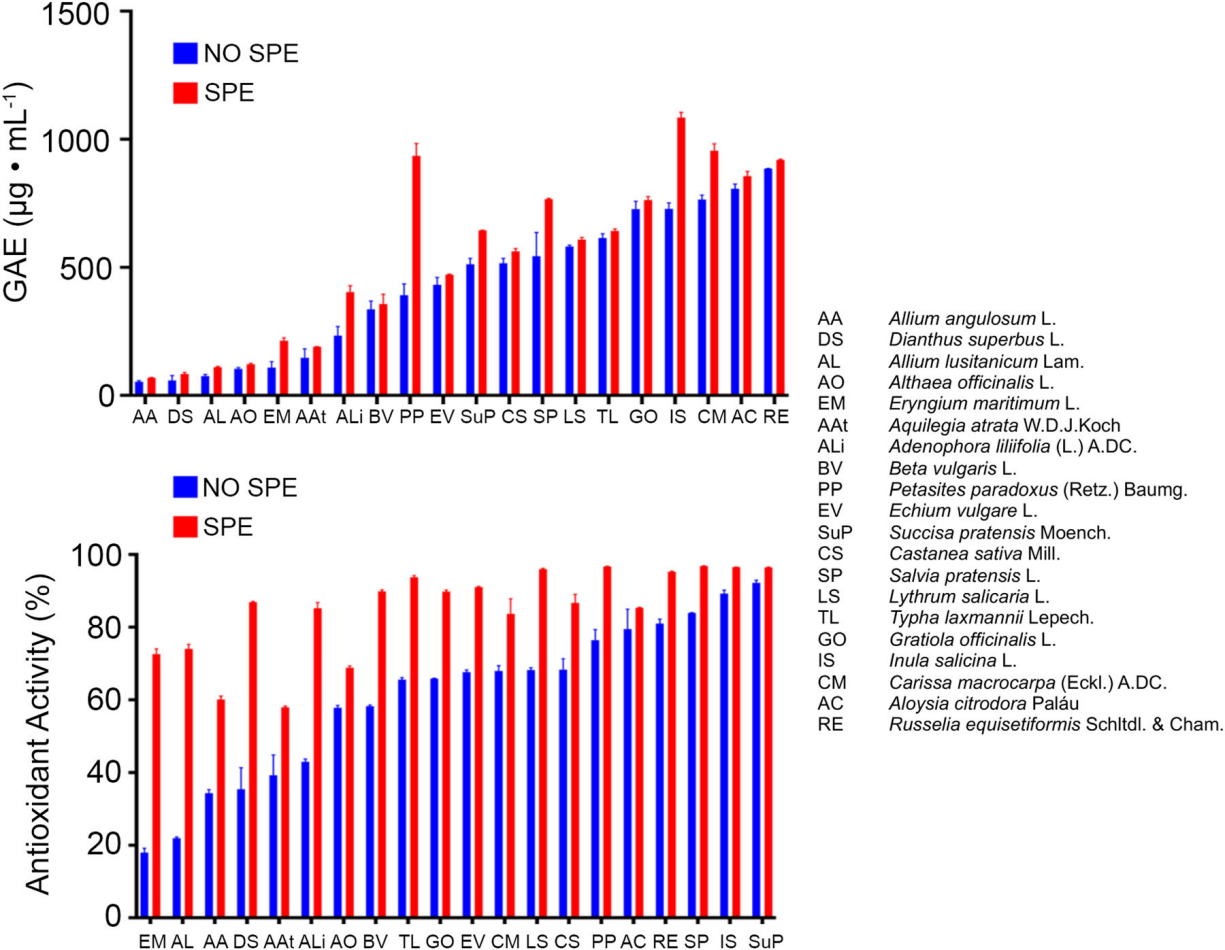
Total phenolic content and antioxidant activity results of 20 treated (red) and untreated (blue) National Biodiversity Future Center extracts. The results are expressed as mean values (mean ± SD). All experiments were performed in triplicate.

After testing the protocols for TPC and antioxidant activity evaluations on *C. avellana*, these protocols were then applied to all 20 NBFC extracts. Overall, TPC and free radical scavenging activity improved for each extract tested (Figure 3). This improvement was dependent on the initial natural matrix and on the initial chlorophyll content, but in general, high TPC was correlated to high antioxidant activity (e.g., *Lithrum salicaria* and *Petasites paradoxus* yielded the best results in both assays). Nevertheless, a few exceptions were observed, as seen for both *Dianthus superbus* and *Allium lusitanicum*, which displayed high antioxidant activity despite low TPC. This discrepancy could be explained by the presence of metabolites belonging to other classes but nonetheless possessing antioxidant activity.

## CONCLUSIONS

This study presents an efficient and sustainable protocol for chlorophyll removal from leaf extracts using polymeric HLB cartridges in an SPE format. The effectiveness of our method stems from its dual‐mode interaction mechanism, simultaneously engaging both the hydrophobic phytol tail and hydrophilic porphyrin head of chlorophylls. Our validation studies demonstrate that the application of this new methodology enables the consistent removal of over 85% of chlorophylls while preserving the phytochemical profiles of the extracts, with particular attention to the phenolic profile. This was confirmed through TPC and antioxidant activity assessment across 20 different taxa. Notably, the protocol also concentrates the polyphenolic content in the final treated extract.

This protocol offers significant practical advantages over traditional methods by simplifying the workflow to a single equilibration step, thereby reducing processing time. This efficiency gain, combined with the capability of parallel sample processing, makes the method ideal for high‐throughput applications in different fields. Thus, it can be useful both to remove chlorophylls for NADD purposes and to concentrate these pigments for use as colors or functional ingredients. Moreover, the flexibility of the protocol is demonstrated through its compatibility with different solvents, although proper attention to loading capacity is essential.

From a sustainability perspective, our method addresses several limitations of existing techniques. Unlike LLE or dispersive activated charcoal approaches, this protocol minimizes solvent consumption and allows for cartridge reuse, significantly reducing waste. Furthermore, the availability of cartridge sizes from 96‐well to 30 cc provides scalability from initial screening to larger‐scale applications. Although throughput is limited by cartridge capacity, this limitation is generally offset by the efficiency of the method and parallel processing capabilities.

In conclusion, this protocol represents a significant advancement, facilitating more reliable bioactivity screening and analytical fingerprinting by providing an efficient, sustainable, and versatile method to eliminate chlorophyll from natural extracts. Its successful validation across diverse taxa and compatibility with standard laboratory equipment make it adaptable to various research settings. The combination of reduced processing time, minimal waste generation, and maintained extract integrity addresses key challenges in natural product research, facilitating more reliable and environmentally conscious drug discovery efforts.

## AUTHOR CONTRIBUTIONS

V.C. and E.M. conceived the research and designed the experiments, and A.F. performed all the experiments. E.M. acquired the funding. All authors wrote and edited the manuscript, and all authors approved the final version of the manuscript.

## Supporting information


**Appendix S1.** Thin‐layer chromatography (TLC) of methanolic *Corylus avellana* extract (no solid‐phase extraction [SPE]), after the SPE purification and chlorophyll fractionation (CHLORO), obtained by applying method B and visualized at 254 nm (A), 366 nm (B), stained with 2,2‐diphenyl‐1‐picrylhydrazyl (DPPH) stain (C), and stained with ceric ammonium molybdate (IV) stain (D). MP: 4Tol/3AcOEt/3MeOH/0.1HOOCH.
**Appendix S2.** TLC chlorophyll removal qualitative analysis of five National Biodiversity Future Center (NBFC) extracts before (NO SPE) and after (SPE) the purification treatment, obtained by applying method A and visualized at 366 nm. (A) *Inula salicina*; (B) *Eryngium maritimum*; (C) *Typha laxmannii*; (D) *Gratiola officinalis*; (E) *Echium vulgare*. MP: 6Hex/1AcOEt/1Acetone/0.4MeOH.
**Appendix S3.** TLC phytochemical qualitative analysis of six NBFC extracts before (NO SPE) and after (SPE) the purification treatment, obtained by applying method B and stained with DPPH stain. (A) *Adenophora liliifolia*, (B) *Gratiola officinalis*, (C) *Althaea officinalis*, (D) *Succisa pratensis*, (E) *Echium vulgare*, (F) *Petasites paradoxus*. MP: 4Tol/3AcOEt/3MeOH/0.1HOOCH.
**Appendix S4.** Overlapped chromatograms of 10 raw NBFC extracts (blue) and treated extracts (red). The analyses were carried out with a UV‐DAD detector and detected at 280 nm (CH 1) and 320 nm (CH 2). (A) *Inula salicina*, (B) *Eryngium maritimum*, (C) *Typha laxmannii*, (D) *Gratiola officinalis*, (E) *Echium vulgare*, (F) *Aquilegia atrata*, (G) *Petasites paradoxus*, (H) *Carissa macrocarpa*, (I) *Adenophora liliifolia*, (J) *Allium angulosum*.
**Appendix S5.** Overlapped total ion chromatograms (TICs) of *Corylus avellana* (A, B) and *Eryngium maritimum* (C, D) raw and treated extracts. Raw extracts are shown in black and treated extracts in blue. Mass spectral analyses were carried out in both negative mode (A, C) and positive mode (B, D). TIC chromatograms were generated in positive and negative ion mode (mass range 100–1000 Da, capillary temperature 220°C, ion spray voltage 3500 V, capillary voltage 120 V, source gas temperature 250°C).
**Appendix S6.** Percentage of chlorophyll removal of both chlorophyll *a* and *b* of *C. avellana* methanolic extract and methanolic NBFC extracts obtained using 1‐cc HLB SPE cartridges and loading 500 µL of extract at 5 mg/mL in methanol. Antioxidant activity and total phenolic count are reported for the same extracts before and after SPE purification to allow for easy comparison and highlight their improvement after SPE.

## Data Availability

All data generated or analyzed during this study are included in this published article and the Supporting Information.

## Appendix 1 Detailed protocol to remove chlorophyll from leaf extracts.

### Materials required

- Waters OASIS HLB cartridges (1 cc and 6 cc) (Waters Corporation, Milford, Massachusetts, USA)
- Vacuum pump (VWR Compact Vacuum Pump For Filtration and Solid Phase Extraction; VWR International, Radnor, Pennsylvania, USA)
- Vacuum manifold to be used for solid‐phase extraction (SPE) purification procedure (Visiprep‐SPE‐vacuum manifold; Supelco/Merck, Darmstadt, Germany)
- Glass tubes suitable for vacuum application
- Conical glass flask suitable for vacuum application and vacuum conical filtration adapters
- Graduated measuring glass cylinder
- Solvents (methanol and acetone)

### Purification procedure (1‐cc cartridge)

1. Initial set up: Plug in the vacuum pump, insert the glass tubes in the manifold, and place the cartridges to be used in their designated place.
2. Cartridge conditioning: Close the vacuum manifold, connect the vacuum tube, and turn on the vacuum pump. Once the system reaches −50 KPa, load 200 μL of clean methanol inside the 1‐cc cartridges. When the methanol has passed through the cartridges, disconnect the tube and turn off the pump. Discard the methanol in the tubes.
3. Purification: Prepare a 5 mg/mL sample of the leaf extract to be purified. Load 500 μL of leaf extract into the cartridges and apply the vacuum. Once purification is complete, transfer the resulting purified extract to a round flask and evaporate under reduced pressure.

### Purification procedure (6‐cc cartridge)

The same procedure is applied when using 6‐cc cartridges, with the loaded volume of extract adjusted based on chlorophyll content.

1. Initial set up: Plug in the vacuum pump, choose the correct vacuum conical filtration adapter to be used for the conical flask, and place the cartridge to be used inside it. This step is crucial, as the adapter must fit inside the neck of the flask and also fit the cartridge well enough to permit vacuum conditions inside the flask.
2. Cartridge conditioning: Apply the vacuum by connecting the vacuum tube and turning on the vacuum pump. Once the system reaches −50 KPa, load 1 mL of clean methanol inside the 6‐cc cartridge. When the methanol has passed through the cartridge, disconnect the tube and turn off the pump. Discard the methanol in the flask.
3. Purification: Prepare a 5 mg/mL sample of the leaf extract to be purified. Using a 10‐mL graduated cylinder measure, load 10 mL of leaf extract into the cartridges and apply the vacuum. Once the purification is complete, transfer the resulting purified extract to a round flask and evaporate under reduced pressure.

### Cartridge recycling

This procedure can be applied to both 1‐cc and 6‐cc cartridges by changing the volume of solvent required for the regeneration.

- 1‐cc cartridges: After the extract purification, load 1 mL of acetone into the used cartridge and then apply the vacuum by connecting the vacuum tube to the manifold and turning on the vacuum pump. At the end of the process, the dark green eluted solution in the glass tubes will be rich in chlorophylls and can be discarded. To obtain efficient chlorophyll purification, the cartridge conditioning step with methanol must be repeated for each recycling cycle.
- 6‐cc cartridges: After the extract purification, using a 10‐mL graduated cylinder, load 6 mL of acetone into the used cartridge and then apply the vacuum by connecting the vacuum tube to the conical flask and turning on the vacuum pump. At the end of the process, the dark green eluted solution in the flask will be rich in chlorophylls and can be discarded. To obtain efficient chlorophyll purification, the cartridge conditioning step with methanol must be repeated for each recycling cycle.

This procedure can be repeated at least five times consecutively, obtaining a satisfactory 80% of chlorophyll *a* and *b* removal at the fifth usage of the same cartridge.

## References

[aps370018-bib-0001] Adumitrăchioaie, A. , M. Tertiș , A. Cernat , R. Săndulescu , and C. Cristea . 2018. Electrochemical methods based on molecularly imprinted polymers for drug detection. A review. International Journal of Electrochemical Science 13(3): 2556–2576.

[aps370018-bib-0002] Agarwal, A. , P. D'Souza , T. S. Johnson , S. M. Dethe , and C. Chandrasekaran . 2014. Use of in vitro bioassays for assessing botanicals. Current Opinion in Biotechnology 25: 39–44. 24484879 10.1016/j.copbio.2013.08.010

[aps370018-bib-0003] Amri, B. , E. Martino , F. Vitulo , F. Corana , L. B. Kaâb , M. Rui , D. Rossi , et al. 2017. *Marrubium vulgare* L. leave [sic] extract: Phytochemical composition, antioxidant and wound healing properties. Molecules 22(11): 1851. 29143793 10.3390/molecules22111851 PMC6150401

[aps370018-bib-0004] Attard, E. 2013. A rapid microtitre plate Folin‐Ciocalteu method for the assessment of polyphenols. Open Life Sciences 8(1): 48–53.

[aps370018-bib-0005] Bahmaei, M. , E. Sadat Sabbaghian , and E. Farzadkish . 2005. Development of a method for chlorophyll removal from canola oil using mineral acids. Journal of the American Oil Chemists' Society 82(9): 679–684.

[aps370018-bib-0006] Bahramsoltani, R. , P. Rostamiasrabadi , Z. Shahpiri , A. M. Marques , R. Rahimi , and M. H. Farzaei . 2018. *Aloysia citrodora* Paláu (Lemon verbena): A review of phytochemistry and pharmacology. Journal of Ethnopharmacology 222: 34–51. 29698776 10.1016/j.jep.2018.04.021

[aps370018-bib-0070] Batlokwa, B. S. , J. Mokgadi , R. Majors , C. Turner , and N. Torto . 2013. A novel molecularly imprinted polymer for the selective removal of chlorophyll from heavily pigmented green plant extracts prior to instrumental analysis. Journal of Chemistry 2013(1): 540240. 10.1155/2013/540240

[aps370018-bib-0007] Bianchini, S. , F. Bovio , S. Negri , F. Guzzo , M. Forcella , and P. Fusi . 2025. *Gratiola officinalis* alcoholic extract targets Warburg effect, apoptosis and cell cycle progression in colorectal cancer cell lines. International Journal of Molecular Sciences 26(5): 2220. 40076837 10.3390/ijms26052220 PMC11900565

[aps370018-bib-0008] Biehler, E. , F. Mayer , L. Hoffmann , E. Krause , and T. Bohn . 2010. Comparison of 3 spectrophotometric methods for carotenoid determination in frequently consumed fruits and vegetables. Journal of Food Science 75(1): C55–C61. 20492150 10.1111/j.1750-3841.2009.01417.x

[aps370018-bib-0009] Bijttebier, S. , E. D'Hondt , S. Apers , N. Hermans , and S. Voorspoels . 2013. Improving method reliability through selective removal of glycerolipid and chlorophyll interferences. Planta Medica 79(13): PH2. 10.1021/jf405477s 24635051

[aps370018-bib-0010] Bijttebier, S. , E. D'Hondt , B. Noten , N. Hermans , S. Apers , and S. Voorspoels . 2014. Tackling the challenge of selective analytical clean‐up of complex natural extracts: The curious case of chlorophyll removal. Food Chemistry 163: 147–153. 24912710 10.1016/j.foodchem.2014.04.098

[aps370018-bib-0011] Blass, B. E. 2015. In vitro screening systems. *In* B. E. Blass [ed.], Basic principles of drug discovery and development, 143–202. Academic Press, Boston, Massachusetts, USA.

[aps370018-bib-0012] Borah, D. , H. Nath , and H. Saikia . 2022. Modification of bentonite clay and its applications: A review. Reviews in Inorganic Chemistry 42(3): 265–282.

[aps370018-bib-0013] Britton, G. , S. Liaaen‐Jensen , and H. Pfander . 1995. Carotenoids, Volume 1A: Isolation and analysis. Birkhäuser, Basel, Switzerland.

[aps370018-bib-0014] Cardellina, J. H. 1983. Step gradient elution in gel permeation chromatography. A new approach to natural products separations. Journal of Natural Products 46(2): 196–199.

[aps370018-bib-0015] Carmichael, J. , W. G. DeGraff , A. F. Gazdar , J. D. Minna , and J. B. Mitchell . 1987. Evaluation of a tetrazolium‐based semiautomated colorimetric assay: Assessment of chemosensitivity testing. Cancer Research 47(4): 936–942. 3802100

[aps370018-bib-0016] Cavalloro, V. , G. Marrubini , R. Stabile , D. Rossi , P. Linciano , G. Gheza , S. Assini , et al. 2021. Microwave‐assisted extraction and HPLC‐UV‐CD determination of (S)‐usnic acid in *Cladonia foliacea* . Molecules 26(2): 455. 33467133 10.3390/molecules26020455 PMC7830470

[aps370018-bib-0017] Cavalloro, V. , N. Marchesi , P. Linciano , D. Rossi , L. I. M. Campagnoli , A. Fossati , K. M. Ahmed , et al. 2024. Neurodegeneration: Can metabolites from *Eremurus persicus* help? Frontiers in Pharmacology 15: e1309766. 10.3389/fphar.2024.1309766 PMC10873958 38370479

[aps370018-bib-0018] Chapman, D. M. 1994. Benefits and limitations of a novel chlorophyll adsorbent. Journal of the American Oil Chemists’ Society 71(4): 397–400.

[aps370018-bib-0019] Commisso, M. , S. Negri , M. Bianconi , S. Gambini , S. Avesani , S. Ceoldo , L. Avesani , and F. Guzzo . 2019. Untargeted and targeted metabolomics and tryptophan decarboxylase in vivo characterization provide novel insight on the development of kiwifruits (*Actinidia deliciosa*). International Journal of Molecular Sciences 20(4): e897. 10.3390/ijms20040897 PMC6413197 30791398

[aps370018-bib-0020] Cooney, D. O. , A. Nagerl , and A. L. Hines . 1983. Solvent regeneration of activated carbon. Water Research 17(4): 403–410.

[aps370018-bib-0021] Cuong, D. X. 2020. Antioxidant chlorophyll purification from maize leaves by liquid‐to‐liquid extraction method. Journal of Drug Delivery and Therapeutics 10(3): 152–158.

[aps370018-bib-0022] Denizot, F. , and R. Lang . 1986. Rapid colorimetric assay for cell growth and survival. Journal of Immunological Methods 89(2): 271–277. 3486233 10.1016/0022-1759(86)90368-6

[aps370018-bib-0023] Donaldson, L. 2020. Autofluorescence in plants. Molecules 25(10): e2393. 10.3390/molecules25102393 PMC7288016 32455605

[aps370018-bib-0024] Ebrahimi, P. , Z. Shokramraji , S. Tavakkoli , D. Mihaylova , and A. Lante . 2023. Chlorophylls as natural bioactive compounds existing in food by‐products: A critical review. Plants 12(7): e1533. 10.3390/plants12071533 PMC10096697 37050159

[aps370018-bib-0025] Farhat, C. , H. Younes , O. A. Alyamani M. Mrad , N. Hourani , H. Khalifeh , Y. El‐Makhour , et al. 2022. Chemical characterization and in vitro biological evaluation of aqueous extract of *Althaea officinalis* L. flower grown in Lebanon. Journal of Herbal Medicine 34: e100575.

[aps370018-bib-0026] Granado, F. , B. Olmedilla , E. Gil‐Martinez , and I. Blanco . 2001. A fast, reliable and low‐cost saponification protocol for analysis of carotenoids in vegetables. Journal of Food Composition and Analysis 14(5): 479–489.

[aps370018-bib-0027] Granata, M. U. , F. Bracco , R. Catoni , V. Cavalloro , and E. Martino . 2021. Secondary metabolites profile and physiological leaf traits in wild and cultivated *Corylus avellana* under different nutritional status. Natural Product Research 35(18): 3100–3107. 31665919 10.1080/14786419.2019.1682577

[aps370018-bib-0028] Jaganjac, M. , V. Sredoja Tisma , and N. Zarkovic . 2021. Short overview of some assays for the measurement of antioxidant activity of natural products and their relevance in dermatology. Molecules 26(17): e5301. 10.3390/molecules26175301 PMC8433703 34500732

[aps370018-bib-0029] Jung, Y. S. , N.‐E. Song , J. Y. Choi , S. H. Hwang , M. Koo , and T. G. Nam . 2022. Evaluation of various clean‐up sorbents in kale followed by LC‐MS/MS analysis of pesticides. Food Science and Biotechnology 31(7): 787–796. 35720463 10.1007/s10068-022-01101-3 PMC9203608

[aps370018-bib-0030] Kai, Z.‐P. , M.‐X. Hou , J.‐J. Zhu , Z.‐P. Jiang , and S. Chen . 2023. Advanced QuEChERS method using core‐shell magnetic molecularly imprinted polymers (Fe_3_O_4_@MIP) for the determination of pesticides in chlorophyll‐rich samples. Foods 12(20): e3742. 10.3390/foods12203742 PMC10606496 37893635

[aps370018-bib-0031] Kim, S. B. , J. Bisson , J. B. Friesen , G. F. Pauli , and C. Simmler . 2020. Selective chlorophyll removal method to “degreen” botanical extracts. Journal of Natural Products 83(6): 1846–1858. 32426979 10.1021/acs.jnatprod.0c00005 PMC7398693

[aps370018-bib-0032] Koetz, M. , L. C. Klein‐Junior , M. C. Santos , T. A. da Silva , N. S. B. Toson , and A. T. Henriques . 2021. An ultrasound assisted extraction–solid‐phase extraction–ultra‐performance liquid chromatography combined strategy for atropine determination in *Atropa belladonna* leaves. Biomedical Chromatography 35(5): e5053. 33314218 10.1002/bmc.5053

[aps370018-bib-0033] Lankatillake, C. , S. Luo , M. Flavel , G. B. Lenon , H. Gill , T. Huynh , and D. A. Dias . 2021. Screening natural product extracts for potential enzyme inhibitors: Protocols, and the standardisation of the usage of blanks in α‐amylase, α‐glucosidase and lipase assays. Plant Methods 17(1): e3. 10.1186/s13007-020-00702-5 PMC7789656 33407662

[aps370018-bib-0034] Larsen, E. , and L. P. Christensen . 2005. Simple saponification method for the quantitative determination of carotenoids in green vegetables. Journal of Agriculture and Food Chemistry 53(17): 6598–6602. 10.1021/jf050622+ 16104772

[aps370018-bib-0035] Li, T. , J. Xu , H. Wu , G. Wang , S. Dai , J. Fan , H. He , and W. Xiang . 2016. A saponification method for chlorophyll removal from microalgae biomass as oil feedstock. Marine Drugs 14(9): e162. 10.3390/md14090162 PMC5039533 27618070

[aps370018-bib-0036] Liang, L. , G. Liu , G. Yu , Y. Song , and Q. Li . 2019. Simultaneous decoloration and purification of crude oligosaccharides from pumpkin (*Cucurbita moschata* Duch) by macroporous adsorbent resin. Food Chemistry 277: 744–752. 30502211 10.1016/j.foodchem.2018.10.138

[aps370018-bib-0037] Makhoukhi, B. , M. A. Didi , D. Villemin , and A. Azzouz . 2009. Acid activation of bentonite for use as a vegetable oil bleaching agent. Grasas y Aceites 60(4): 343–349.

[aps370018-bib-0038] Markman, M. 1991. Taxol: An important new drug in the management of epithelial ovarian cancer. Yale Journal of Biology and Medicine 64(6): 583–590. 1687343 PMC2589424

[aps370018-bib-0039] Martino, E. , S. Della Volpe , E. Terribile , E. Benetti , M. Sakaj , A. Centamore , A. Sala , and S. Collina . 2017. The long story of camptothecin: From traditional medicine to drugs. Bioorganic and Medicinal Chemistry Letters 27(4): 701–707. 28073672 10.1016/j.bmcl.2016.12.085

[aps370018-bib-0040] Martino, E. , M. Tarantino , M. Bergamini , V. Castelluccio , A. Coricello , M. Falcicchio , E. Lorusso , and S. Collina . 2019. Artemisinin and its derivatives: Ancient tradition inspiring the latest therapeutic approaches against malaria. Future Medicinal Chemistry 11(12): 1443–1459. 31298579 10.4155/fmc-2018-0337

[aps370018-bib-0041] Martins, T. , A. Novo Barros , E. Rosa , and L. Antunes . 2023. Enhancing health benefits through chlorophylls and chlorophyll‐rich agro‐food: A comprehensive review. Molecules 28(14): e5344. 10.3390/molecules28145344 PMC10384064 37513218

[aps370018-bib-0042] Mokaya, R. , W. Jones , M. E. Davies , and M. E. Whittle . 1994. The mechanism of chlorophyll adsorption on acid‐activated clays. Journal of Solid State Chemistry 111(1): 157–163.

[aps370018-bib-0043] Nebergall, R. S. , D. R. Taylor , and C. J. Kucharz . 1994. Process for regenerating spent acid‐activated bentonite clays and smectite catalysts. U.S. Patent No. 5,358,915 [issued 25 October 1994].

[aps370018-bib-0044] Newman, D. J. , and G. M. Cragg . 2020. Natural products as sources of new drugs over the nearly four decades from 01/1981 to 09/2019. Journal of Natural Products 83(3): 770–803. 32162523 10.1021/acs.jnatprod.9b01285

[aps370018-bib-0045] Oberhuber, M. , J. Berghold , K. Breuker , S. Hörtensteiner , and B. Kraütler . 2003. Breakdown of chlorophyll: A nonenzymatic reaction accounts for the formation of the colorless “nonfluorescent” chlorophyll catabolites. Proceedings of the National Academy of Sciences, USA 100(12): 6910–6915. 10.1073/pnas.1232207100 PMC165803 12777622

[aps370018-bib-0046] Pegan, S. D. , Y. Tian , V. Sershon , and A. D. Mesecar . 2010. A universal, fully automated high throughput screening assay for pyrophosphate and phosphate release from enzymatic reactions. Combinatorial Chemistry and High Throughput Screening 13(1): 27–38. 20201823 10.2174/138620710790218203

[aps370018-bib-0047] Pellavio, G. , M. Rui , L. Caliogna , E. Martino , G. Gastaldi , S. Collina , and U. Laforenza . 2017. Regulation of aquaporin functional properties mediated by the antioxidant effects of natural compounds. International Journal of Molecular Sciences 18(12): e2665. 10.3390/ijms18122665 PMC5751267 29292793

[aps370018-bib-0048] Phaisan, S. , G. Yusakul , A. Sakdamas , N. Taluengjit , S. Sakamoto , and W. Putalun . 2020. A green and effective method using oils to remove chlorophyll from *Chromolaena odorata* (L.) R.M. King & H. Rob. Songklanakarin Journal of Science and Technology 42(5): 1084–1090.

[aps370018-bib-0049] Porra, R. J. , W. A. Thompson , and P. E. Kriedemann . 1989. Determination of accurate extinction coefficients and simultaneous equations for assaying chlorophylls *a* and *b* extracted with four different solvents: Verification of the concentration of chlorophyll standards by atomic absorption spectroscopy. Biochimica et Biophysica Acta ‐ Bioenergetics 975(3): 384–394.

[aps370018-bib-0050] Prinsloo, G. , G. Papadi , M. G. Hiben , L. de Haan , J. Louisse , K. Beekmann , J. Vervoort , and I. M. C. M. Rietjens . 2017. In vitro bioassays to evaluate beneficial and adverse health effects of botanicals: Promises and pitfalls. Drug Discovery Today 22(8): 1187–1200. 28533190 10.1016/j.drudis.2017.05.002

[aps370018-bib-0051] Raks, V. , H. Al‐Suod , and B. Buszewski . 2018. Isolation, separation, and preconcentration of biologically active compounds from plant matrices by extraction techniques. Chromatographia 81(2): 189–202. 29449742 10.1007/s10337-017-3405-0 PMC5807477

[aps370018-bib-0052] Rawa‐Adkonis, M. , L. Wolska , A. Przyjazny , and J. Namieśnik . 2006. Sources of errors associated with the determination of PAH and PCB analytes in water samples. Analytical Letters 39(11): 2317–2331.

[aps370018-bib-0053] Rosa, M. E. , A. M. Ferreira , C. M. S. S. Neves , M. R. Almeida , R. Barros , A. C. Cristovão , A. C. A. Sousa , et al. 2023. Valorisation of red beet waste: One‐step extraction and separation of betalains and chlorophylls using thermoreversible aqueous biphasic systems. Green Chemistry 25(5): 1852–1864.

[aps370018-bib-0054] Sabah, E. 2007. Decolorization of vegetable oils: Chlorophyll‐*a* adsorption by acid‐activated sepiolite. Journal of Colloid and Interface Science 310(1): 1–7. 17300794 10.1016/j.jcis.2007.01.044

[aps370018-bib-0055] Sajini, T. , and B. Mathew . 2021. A brief overview of molecularly imprinted polymers: Highlighting computational design, nano and photo‐responsive imprinting. Talanta Open 4: e100072.

[aps370018-bib-0056] Samat, N. , M. F. Ng , N. F. Ruslan , K. S. Okuda , P. J. Tan , and V. Patel . 2018. Interference potential of tannins and chlorophylls in zebrafish phenotypic‐based assays. ASSAY and Drug Development Technologies 16(7): 408–419. 29985634 10.1089/adt.2017.833

[aps370018-bib-0057] Scheer, H. 2022. Chlorophylls: A personal snapshot. Molecules 27(3): e1093. 10.3390/molecules27031093 PMC8838077 35164358

[aps370018-bib-0058] Shi, Y. , T. Liu , Y. Han , X. Zhu , X. Zhao , X. Ma , D. Jiang , and Q. Zhang . 2017. An efficient method for decoloration of polysaccharides from the sprouts of *Toona sinensis* (A. Juss.) Roem by anion exchange macroporous resins. Food Chemistry 217: 461–468. 27664659 10.1016/j.foodchem.2016.08.079

[aps370018-bib-0059] Soares, S. , T. Rosado , M. Barroso , and E. Gallardo . 2023. Solid phase‐based microextraction techniques in therapeutic drug monitoring. Pharmaceutics 15(4): e1055. 10.3390/pharmaceutics15041055 PMC10142207 37111541

[aps370018-bib-0060] Srivastava, N. , and M. Kuddus . 2019. Use of ion‐exchange resin in reactive separation. *In* T. A. R. Inamuddin and A. M. Asiri [eds.], Applications of ion exchange materials in chemical and food industries, 125–137. Springer International Publishing, Cham, Switzerland.

[aps370018-bib-0061] Tang, D. Y. Y. , K. W. Chew , F. G. Gentili , T. A. Kurniawan , Y.‐K. Park , H. S. H. Munawaroh , S. Rajendran , et al. 2023. Performance of bleaching clays in dechlorophyllisation of microalgal oil: A comparative study. Process Biochemistry 129: 94–101.

[aps370018-bib-0062] Tzima, K. , N. P. Brunton , and D. K. Rai . 2020. Evaluation of the impact of chlorophyll removal techniques on polyphenols in rosemary and thyme by‐products. Journal of Food Biochemistry 44(3): e13148. 31962370 10.1111/jfbc.13148

[aps370018-bib-0063] United Chemical Technologies . 2024. ChloroFiltr. Website https://www.unitedchem.com/product/chlorofiltr/ (accessed 27 June 2025).

[aps370018-bib-0064] Van der Vegt, M. , R. Kause , B. Berendsen , and S. Van Leeuwen . 2022. Dispersive solid‐phase extraction and solid‐phase extraction for ppt‐level PFAS analysis in apples: A comparison. LCGC Supplements 35(S7): 25–27.

[aps370018-bib-0065] Van Nieuwerburgh, F. C. W. , S. R. F. Vande Casteele , L. Maes , A. Goossens , D. Inzé , J. Van Bocxlaer , and D. L. D. Deforce . 2006. Quantitation of artemisinin and its biosynthetic precursors in *Artemisia annua* L. by high performance liquid chromatography–electrospray quadrupole time‐of‐flight tandem mass spectrometry. Journal of Chromatography A 1118(2): 180–187. 16650427 10.1016/j.chroma.2006.03.121

[aps370018-bib-0066] Vaz, B. M. C. , M. Martins , L. M. de Souza Mesquita , M. C. Neves , A. P. M. Fernandes , D. C. G. A. Pinto , M. G. P. M. S. Neves , et al. 2022. Using aqueous solutions of ionic liquids as chlorophyll eluents in solid‐phase extraction processes. Chemical Engineering Journal 428: e131073.

[aps370018-bib-0067] Vergara‐Luis, I. , J. C. Báez‐Millán , I. Baciero , B. González‐Gaya , M. Olivares , O. Zuloaga , and A. Prieto . 2023. Comparison of conventional and dispersive solid phase extraction clean‐up approaches for the simultaneous analysis of tetracyclines and sulfonamides in a variety of fresh vegetables. Talanta 254: e124192. 10.1016/j.talanta.2022.124192 36527910

[aps370018-bib-0068] Weathers, P. J. , P. R. Arsenault , P. S. Covello , A. McMickle , K. H. Teoh , and D. W. Reed . 2011. Artemisinin production in *Artemisia annua*: Studies *in planta* and results of a novel delivery method for treating malaria and other neglected diseases. Phytochemical Reviews 10(2): 173–183. 10.1007/s11101-010-9166-0 PMC3106422 21643453

[aps370018-bib-0069] Yao, L. , Y. Jiang , N. Datta , R. Singanusong , X. Liu , J. Duan , K. Raymont , et al. 2004. HPLC analyses of flavanols and phenolic acids in the fresh young shoots of tea (*Camellia sinensis*) grown in Australia. Food Chemistry 84(2): 253–263.

