# Supplementary material for: A practical and easy‐to‐scale protocol for removing chlorophylls from leaf extracts
Source: Appl Plant Sci. 2025 Jul 29;13(4):e70018. doi: 10.1002/aps3.70018 (PMC12319715; doi:10.1002/aps3.70018)
Supplement: Supplementary file 1 — Appendix S1. Thin‐layer chromatography (TLC) of methanolic Corylus avellana extract (no solid‐phase extraction [SPE]), after the SPE purification and chlorophyll fractionation (CHLORO), obtained by applying method B and visualized at 254 nm (A), 366 nm (B), stained with 2,2‐diphenyl‐1‐picrylhydrazyl (DPPH) stain (C), and stained with ceric ammonium molybdate (IV) stain (D). MP: 4Tol/3AcOEt/3MeOH/0.1HOOCH. Appendix S2. TLC chlorophyll removal qualitative analysis of five National Biodiversity Future Center (NBFC) extracts before (NO SPE) and after (SPE) the purification treatment, obtained by applying method A and visualized at 366 nm. (A) Inula salicina; (B) Eryngium maritimum; (C) Typha laxmannii; (D) Gratiola officinalis; (E) Echium vulgare. MP: 6Hex/1AcOEt/1Acetone/0.4MeOH. Appendix S3. TLC phytochemical qualitative analysis of six NBFC extracts before (NO SPE) and after (SPE) the purification treatment, obtained by applying method B and stained with DPPH stain. (A) Adenophora liliifolia, (B) Gratiola officinalis, (C) Althaea officinalis, (D) Succisa pratensis, (E) Echium vulgare, (F) Petasites paradoxus. MP: 4Tol/3AcOEt/3MeOH/0.1HOOCH. Appendix S4. Overlapped chromatograms of 10 raw NBFC extracts (blue) and treated extracts (red). The analyses were carried out with a UV‐DAD detector and detected at 280 nm (CH 1) and 320 nm (CH 2). (A) Inula salicina, (B) Eryngium maritimum, (C) Typha laxmannii, (D) Gratiola officinalis, (E) Echium vulgare, (F) Aquilegia atrata, (G) Petasites paradoxus, (H) Carissa macrocarpa, (I) Adenophora liliifolia, (J) Allium angulosum. Appendix S5. Overlapped total ion chromatograms (TICs) of Corylus avellana (A, B) and Eryngium maritimum (C, D) raw and treated extracts. Raw extracts are shown in black and treated extracts in blue. Mass spectral analyses were carried out in both negative mode (A, C) and positive mode (B, D). TIC chromatograms were generated in positive and negative ion mode (mass range 100–1000 Da, capillary temperature 220 [file APS3-13-e70018-s001.pdf]

DOI 10.1002/aps3.70018

Supporting Information for "A practical and easy-to-scale protocol for removing chlorophylls from leaf extracts"

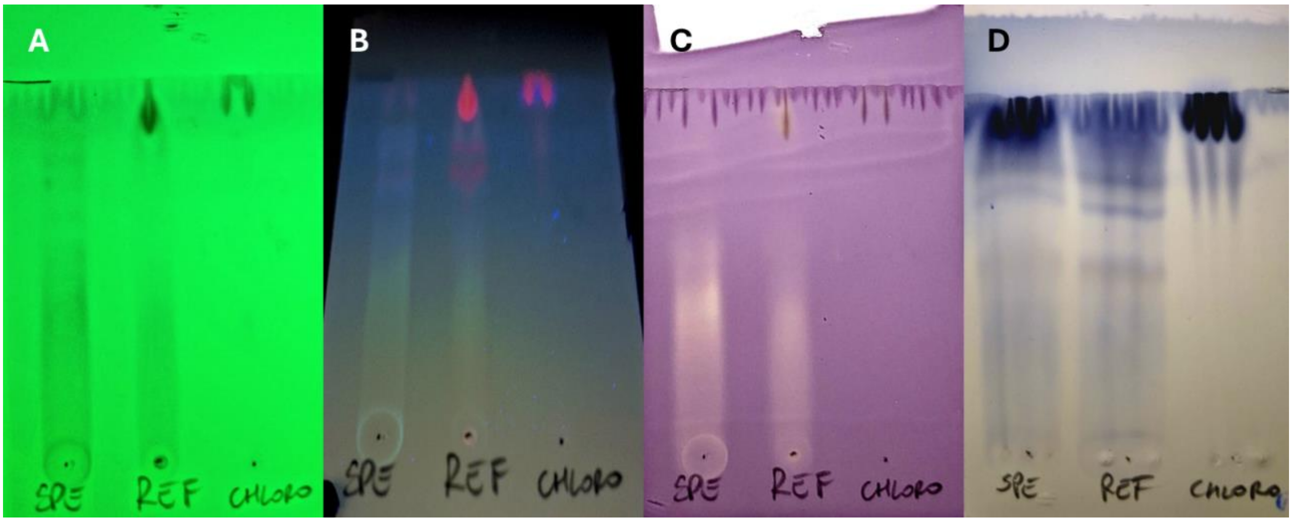

Appendix S1. Thin-layer chromatography (TLC) of methanolic *Corylus avellana* extract (no solid-phase extraction [SPE]), after the SPE purification and chlorophyll fractionation (CHLORO), obtained by applying method B and visualized at 254 nm (A), 366 nm (B), stained with 2,2-diphenyl-1-picrylhydrazyl (DPPH) stain (C), and stained with ceric ammonium molybdate (IV) stain (D). MP: 4Tol/3AcOEt/3MeOH/0.1HOOCH.

DOI 10.1002/aps3.70018

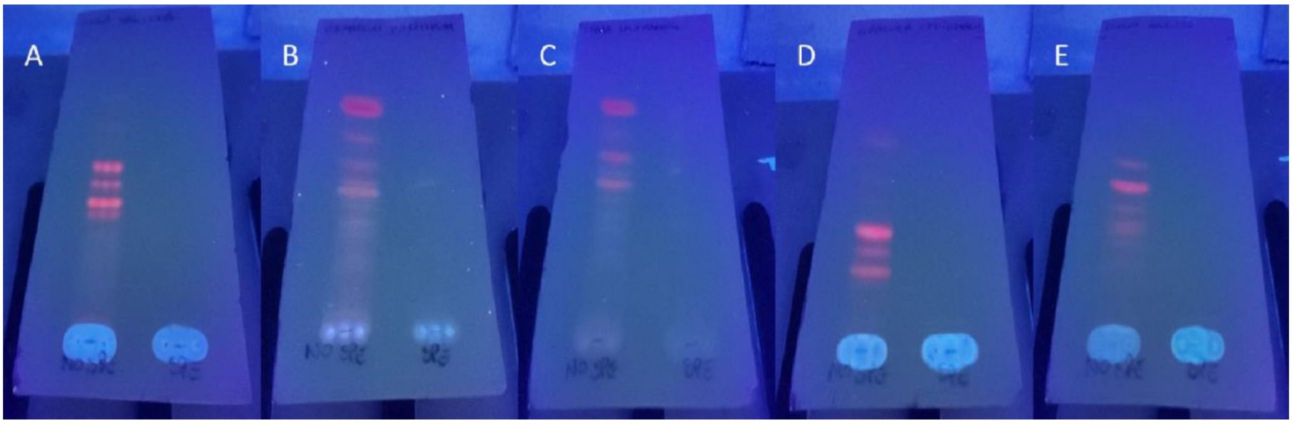

Appendix S2. TLC chlorophyll removal qualitative analysis of five National Biodiversity Future Center (NBFC) extracts before (NO SPE) and after (SPE) the purification treatment, obtained by applying method A and visualized at 366 nm. (A) *Inula salicina*; (B) *Eryngium maritimum*; (C) *Typha laxmannii*; (D) *Gratiola officinalis*; (E) *Echium vulgare*. MP: 6Hex/1AcOEt/1Acetone/0.4MeOH.

DOI 10.1002/aps3.70018

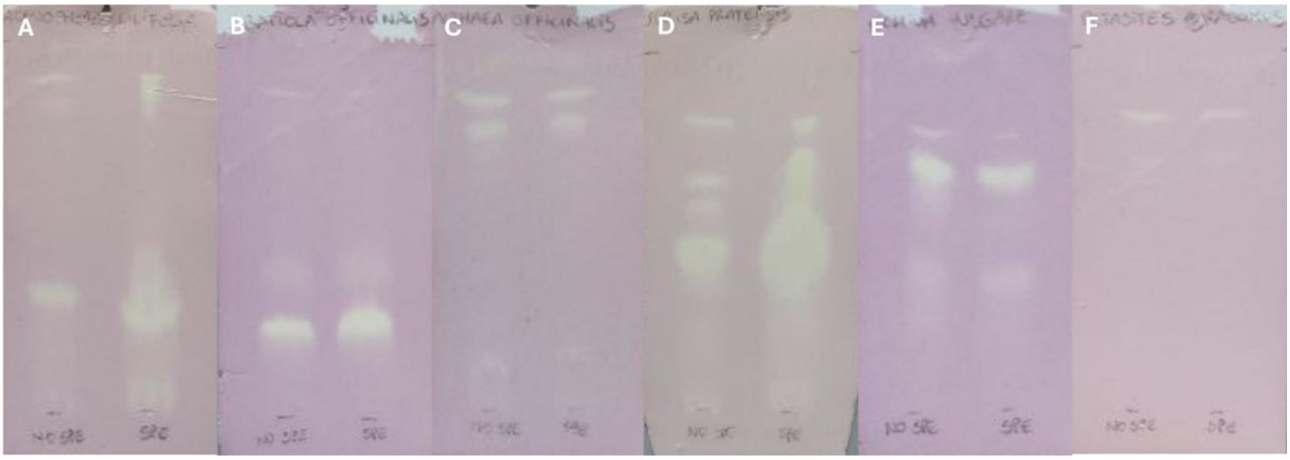

Appendix S3. TLC phytochemical qualitative analysis of six NBFC extracts before (NO SPE) and after (SPE) the purification treatment, obtained by applying method B and stained with DPPH stain. (A) *Adenophora liliifolia*, (B) *Gratiola officinalis*, (C) *Althaea officinalis*, (D) *Succisa pratensis*, (E) *Echium vulgare*, (F) *Petasites paradoxus*. MP: 4Tol/3AcOEt/3MeOH/0.1HOOCH.

DOI 10.1002/aps3.70018

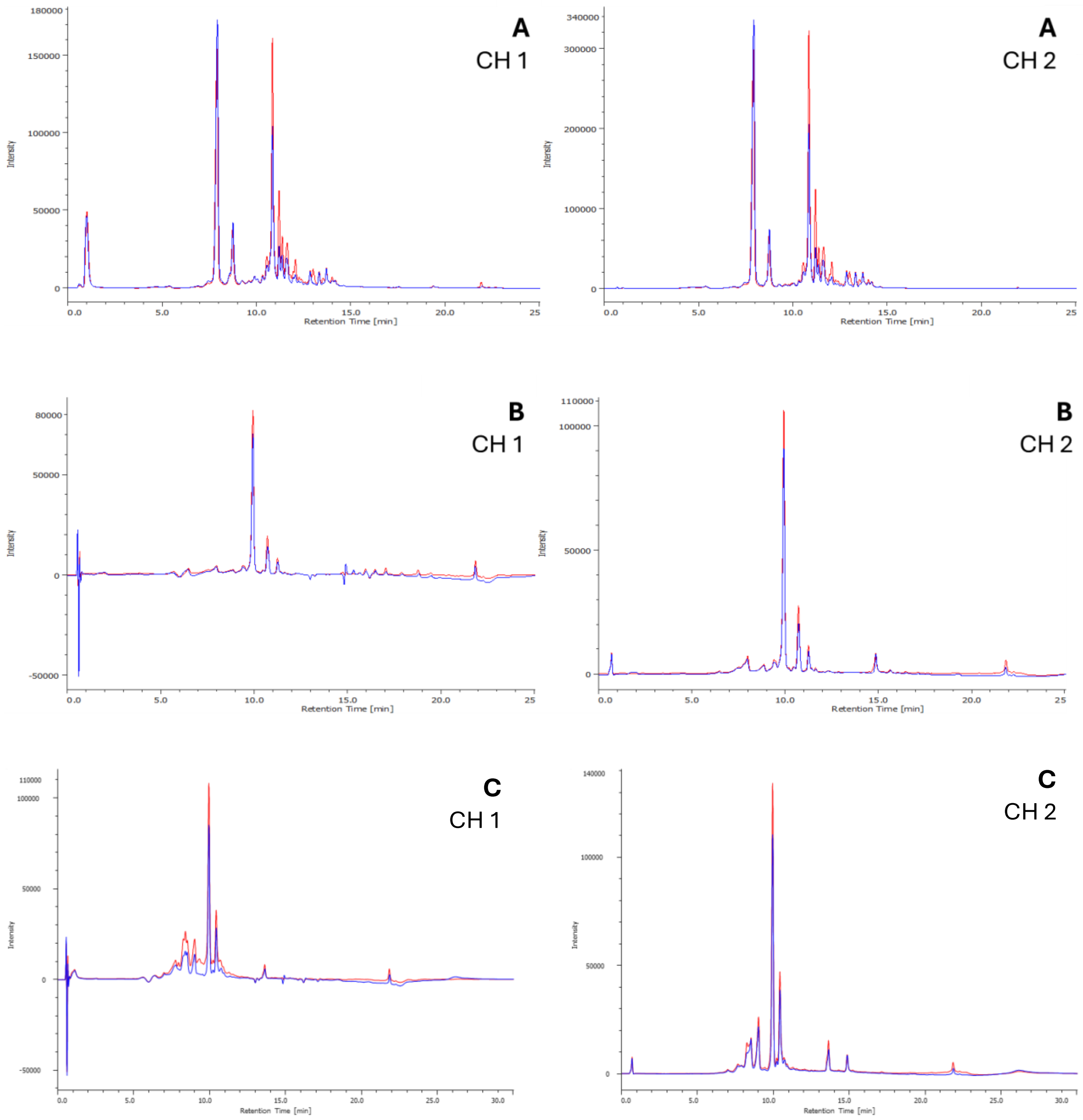

DOI 10.1002/aps3.70018

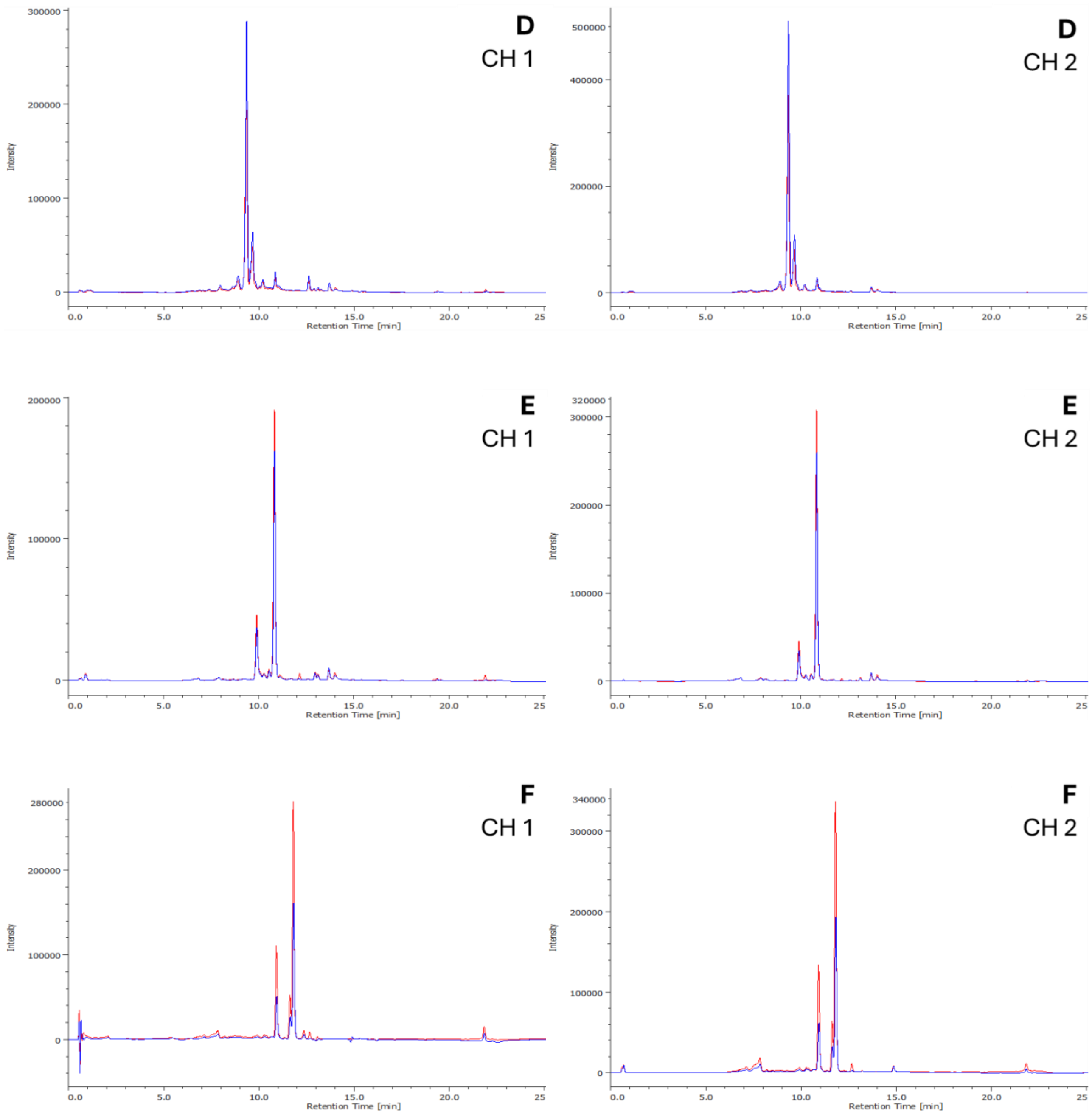

DOI 10.1002/aps3.70018

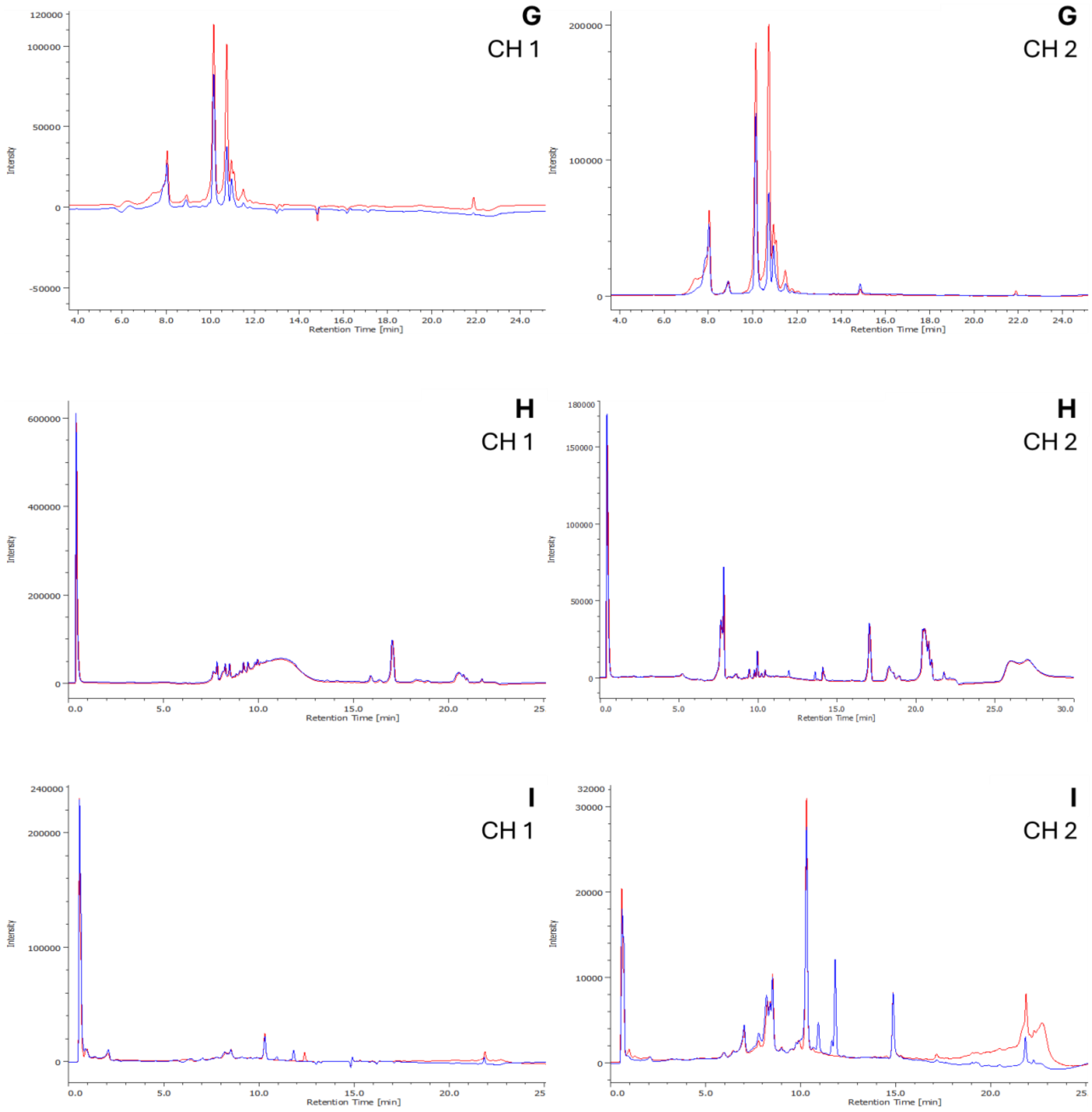

DOI 10.1002/aps3.70018

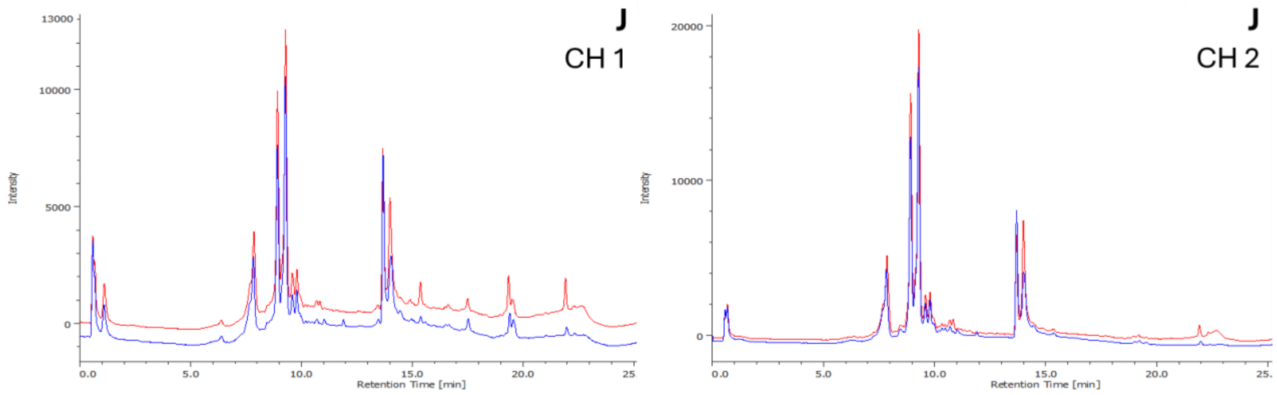

Appendix S4. Overlapped chromatograms of 10 raw NBFC extracts (blue) and treated extracts (red). The analyses were carried out with a UV-DAD detector and detected at 280 nm (CH 1) and 320 nm (CH 2). (A) *Inula salicina*, (B) *Eryngium maritimum*, (C) *Typha laxmannii*, (D) *Gratiola officinalis*, (E) *Echium vulgare*, (F) *Aquilegia atrata*, (G) *Petasites paradoxus*, (H) *Carissa macrocarpa*, (I) *Adenophora liliifolia*, (J) *Allium angulosum.*

HPLC analyses were performed with a Jasco system (PU-4180 pump, AS-4050 autosampler, MD-4010 photo diode array detector, CO-4060 column oven, and LC-Net/ADC interface box) and ChromeNav Ver.II HPLC software. The analyses were carried out at 0.35 mL/min flow rate, using a Acquity BEH C18 (100 × 2.1 mm, pores 1.7 µm) column and MilliQ water + 0.1% formic acid and acetonitrile as solvents in gradient.

Time [min]	% of water + 0.1% formic acid	% acetonitrile
1	99	1
10	60	40
13.5	30	70
15	10	90
16.5	0	100
20	0	100
20.1	99	1
30	99	1

DOI 10.1002/aps3.70018

HPLC-MS were performed with a Jasco HPLC system (PU-4185 binary semi-micro pump, AS-4050 autosampler, MD-2010Plus multiwavelength detector) combined with a Mass Spectrometer (Advion expressionS), ChromeNav Ver.II HPLC software and AdvionCheMS Express MS software. The analyses were carried out at 0.8 mL/min flow rate, using a Purospher STARP C18 (150 × 4.6 mm, pores 5 µm) column and MilliQ water + 0.05% formic acid and acetonitrile + 0.05% formic acid as solvents in gradient.

Time [min]	% of water + 0.05% formic acid	% acetonitrile + 0.05% formic acid
0.1	85	15
5	70	30
9	55	45
12	48	52
14	0	100
15	85	15
25	85	15

DOI 10.1002/aps3.70018

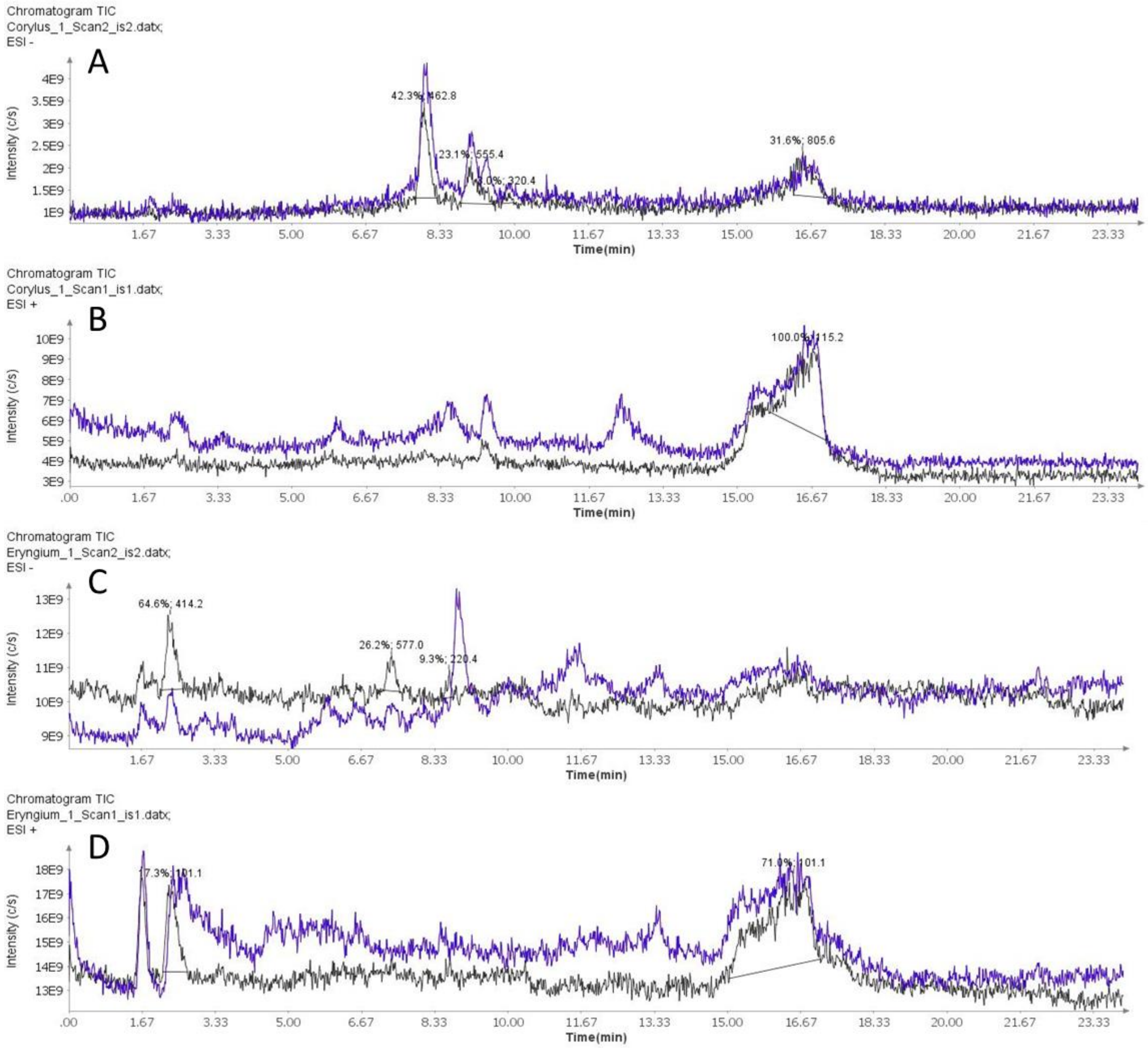

Appendix S5. Overlapped total ion chromatogram (TICs) of *Corylus avellana* (A, B) and *Eryngium maritimum* (C, D) raw and treated extracts. Raw extracts are shown in black and treated extracts in blue. Mass spectral analyses were carried out in both negative mode (A, C) and positive mode (B, D). TICs were generated in positive and negative ion mode (mass range 100–1000 Da, capillary temperature 220°C, ion spray voltage 3500 V, capillary voltage 120 V, source gas temperature 250°C).

DOI 10.1002/aps3.70018

Taxa	Chlorophyll *a* % removal	Chlorophyll *b* % removal	Pre - SPE		Post - SPE	
			Antioxidant activity (%)	TPC (GAE µg·mL^{-1})	Antioxidant activity (%)	TPC (GAE µg·mL^{-1})
Corylus avellana L.	85% ± 0.3	90% ± 0.1	79% ± 8.5	790 ± 12.1	83% ± 2.9	1348 ± 13.4
Adenophora liliifolia (L.) A.DC.	88% ± 0.2	86% ± 0.6	43% ± 1.4	544 ± 13.0	85% ± 2.7	767 ± 3.5
Allium angulosum L.	93% ± 0.8	96% ± 0.3	34% ± 1.6	148 ± 4.8	60% ± 1.6	190 ± 1.4
Allium lusitanicum Lam.	85% ± 1.1	89% ± 0.8	22% ± 0.6	392 ± 6.1	74% ± 2.1	935 ± 69.2
Aloysia citrodora Paláu	91% ± 0.5	88% ± 0.7	80% ± 9.4	336 ± 46.6	85% ± 0.2	357 ± 53.7
Althaea officinalis L.	93% ± 1.3	87% ± 0.9	58% ± 1.1	516 ± 26	69% ± 0.8	563 ± 14.1
Aquilegia atrata W.D.J.Koch	93% ± 0.4	85% ± 0.7	39% ± 9.7	886 ± 2.1	58% ± 0.6	919 ± 4.2
Beta vulgaris L.	88% ± 0.8	82% ± 1.2	58% ± 0.7	59 ± 2.8	90% ± 0.8	84 ± 9.2
Castanea sativa Mill.	92% ± 0.7	88% ± 0.3	68% ± 5.1	109 ± 3.3	87% ± 4	214 ± 15.5
Dianthus superbus L.	91% ± 1.1	86% ± 0.8	36% ± 10.2	728 ± 32.3	87% ± 0.4	1084 ± 28.9
Echium vulgare L.	86% ± 0.2	84% ± 0.3	68% ± 1.1	432 ± 41.2	91% ± 0.4	471 ± 2.8

DOI 10.1002/aps3.70018

Eryngium maritimum L.	83% ± 1.4	88% ± 1.2	18% ± 2.1	104 ± 7.1	73% ± 2.5	122 ± 4.9
Gratiola officinalis L.	87% ± 0.4	90% ± 0.9	66% ± 0.2	727 ± 43.1	90% ± 0.7	763 ± 19.8
Inula salicina L.	83% ± 0.1	89% ± 0.5	89% ± 1.7	765 ± 24.1	97% ± 0.1	955 ± 38.2
Lythrum salicaria L.	81% ± 0.8	87% ± 0.3	68.2 ± 1.2	76 ± 9.2	96% ± 0.3	111 ± 3.5
Petasites paradoxus (Retz.) Baumg.	93% ± 1.3	88% ± 0.7	76% ± 5.1	582 ± 7.8	97% ± 0.1	609 ± 12
Russelia equisetiformis Schltdl. & Cham.	96% ± 0.2	98% ± 0.5	81% ± 2.2	54 ± 6.4	95% ± 0.2	69 ± 1.4
Salvia pratensis L.	86% ± 0.7	92% ± 0.4	84% ± 0.2	234 ± 5.2	97% ± 0.1	404 ± 34.6
Succisa pratensis Moench	85% ± 0.9	83% ± 1.3	92% ± 1.3	512 ± 32.5	96% ± 0.3	644.5 ± 0.7
Typha laxmannii Lepech.	91% ± 0.8	93% ± 0.3	66% ± 1.1	615 ± 23.4	94% ± 0.8	642 ± 11.3
Carissa macrocarpa (Eckl.) A.DC.	84% ± 1.2	93% ± 0.7	68% ± 9.7	807 ± 25.5	84% ± 7.1	856 ± 25.5

Appendix S6. Percentage of chlorophyll removal of both chlorophyll *a* and *b* of *C. avellana* methanolic extract and methanolic NBFC extracts obtained using 1-cc HLB SPE cartridges and loading 500 µL of extract at 5 mg/mL in methanol. Antioxidant activity and total phenolic count are reported for the same extracts before and after SPE purification to allow for easy comparison and highlight their improvement after SPE.
